# Relationship with the diaphragm to predict the surgical outcome in large and giant pituitary adenomas

**DOI:** 10.3389/fsurg.2022.962709

**Published:** 2022-09-01

**Authors:** Ethan Harel, Giulia Cossu, Roy Thomas Daniel, Mahmoud Messerer

**Affiliations:** Department of Neurosurgery, University Hospital of Lausanne and University of Lausanne, Lausanne, Switzerland

**Keywords:** cavernous sinus invasion, diaphragm, endoscopy, pituitary adenoma, surgery

## Abstract

**Objective:**

Large and giant pituitary adenomas (L- and G-PAs) continue to remain a surgical challenge. The diaphragm may have a role in determining the shape of the tumor and therefore influencing the extent of resection. Our study aims to analyze our surgical series of L- and G-PAs according to their relationship with the diaphragm and invasion of cavernous sinus (CS).

**Material and methods:**

We performed a retrospective analysis of our surgical series of patients operated for L- and G-PAs. We categorized the tumors into four grades according to their relationship with the diaphragm: grade 1 (supradiaphragmatic component with a wide incompetent diaphragm), grade 2 (purely infra-diaphragmatic tumor with a competent diaphragm), grade 3 (dumbbell-shape tumors), and grade 4 (multilobulated tumor with invasion of the subarachnoid space).

**Results:**

A total of 37 patients were included in our analysis. According to our classification, 43.3% of patients had grade 1 tumors, 27% had grade 2, 5.4% had grade 3, and 24.3% had grade 4 tumors. CS invasion was confirmed intraoperatively in 17 out of 37 patients (46%). The gross total resection (GTR) was obtained in 19% of the cases, near-total resection in 46%, and subtotal resection in 35%. All the patients who achieved GTR had grade 1 tumors and the lowest rate of CS invasion (*p* < 0.01).

**Conclusion:**

Radiological evaluation of the tumor relationship with the diaphragm, invasion of CS, and invasion of the subarachnoid space are crucial to plan the surgical strategy and maximize the possibilities of achieving GTR in L- and G-PAs.

## Introduction

Pituitary adenomas (PAs) account for approximately 10% of intracranial neoplasms, are the third most common tumor, and account for more than 90% of pituitary tumors (1–4).

Tumor size and invasion of surrounding structures remain the important factors in the prediction of the extent of resection. Large PAs (L-PAs) are defined as tumors with a maximal diameter of ≥30 mm, while giant PAs (G-PAs) are tumors with a maximal diameter of ≥40 mm (5–7). These tumors account for 6%–10% of PAs in recent surgical series (3, 8). In most cases, L-PAs and G-PAs are non-functional tumors (6, 9) and are diagnosed because of their mass effect on the optic pathways, the normal pituitary gland, or more rarely, their invasion of the cavernous sinus. In these situations, besides prolactin-secreting adenomas, surgery remains the first choice of treatment.

The main factors that will influence the extent of resection with large and giant lesions are the invasion of the cavernous sinus (7, 18, 19), the invasion of the subarachnoid space with encasement of the arteries of Willis circle, the optic/oculomotor nerves in their cisternal portion, and last but not the least, the consistency of the adenoma (10, 17, 20, 21). Although intuitive, tumor shape plays an important role, as tumors with a multicompartmental morphology and invasion of neurovascular with structures still represent a surgical challenge when compared with tumors of similar size but with a more regular shape. The former is associated with a more limited extent of resection and a higher risk of complications (5, 17).

The diaphragm is the dural sheath that separates the sella turcica from the chiasmatic cistern, leaving just an opening for the pituitary stalk (22, 23). The size and configuration of this opening may vary remarkably from 3 mm to 13 mm antero-posteriorly and from 3 mm to 15 mm on the lateral axis, physiologically (22–24). These variations in length and width might be the factors in determining the shape of the PA, and we can hypothesize that the competency of the diaphragm may influence the shape of the tumor and consequently the extent of resection.

Our study aims to analyze our surgical series of L-PAs and G-PAs according to their relationship with the diaphragm and invasion of CS and describe if the extent of resection can be predicted based on these factors.

## Materials and methods

We performed a retrospective analysis of our consecutive surgical series on patients operated for PA between June 2011 and April 2020, and we extracted all tumors with a maximal diameter of ≥30 mm. They were classified into two types: large (30–39 mm, L-PAs) and giant (≥40 mm, G-PAs) tumors. Other tumors besides PAs arising from the sellar region or the pituitary stalk were excluded.

All patients were evaluated in the preoperative period using cerebral 1.5 or 3T MRI scanners (all Siemens, Erlangen, Germany) with 1.5-mm thick slices (or 2 mm on 1.5T scanners) with unenhanced sagittal T1-weighted spin-echo, coronal T2-weighted, dynamic coronal T1-weighted spin echo, and enhanced sagittal and coronal T1-weighted spin-echo sequences after gadolinium injection. An MRI with similar sequences was performed again 3 months after surgery.

**Figure 1:**
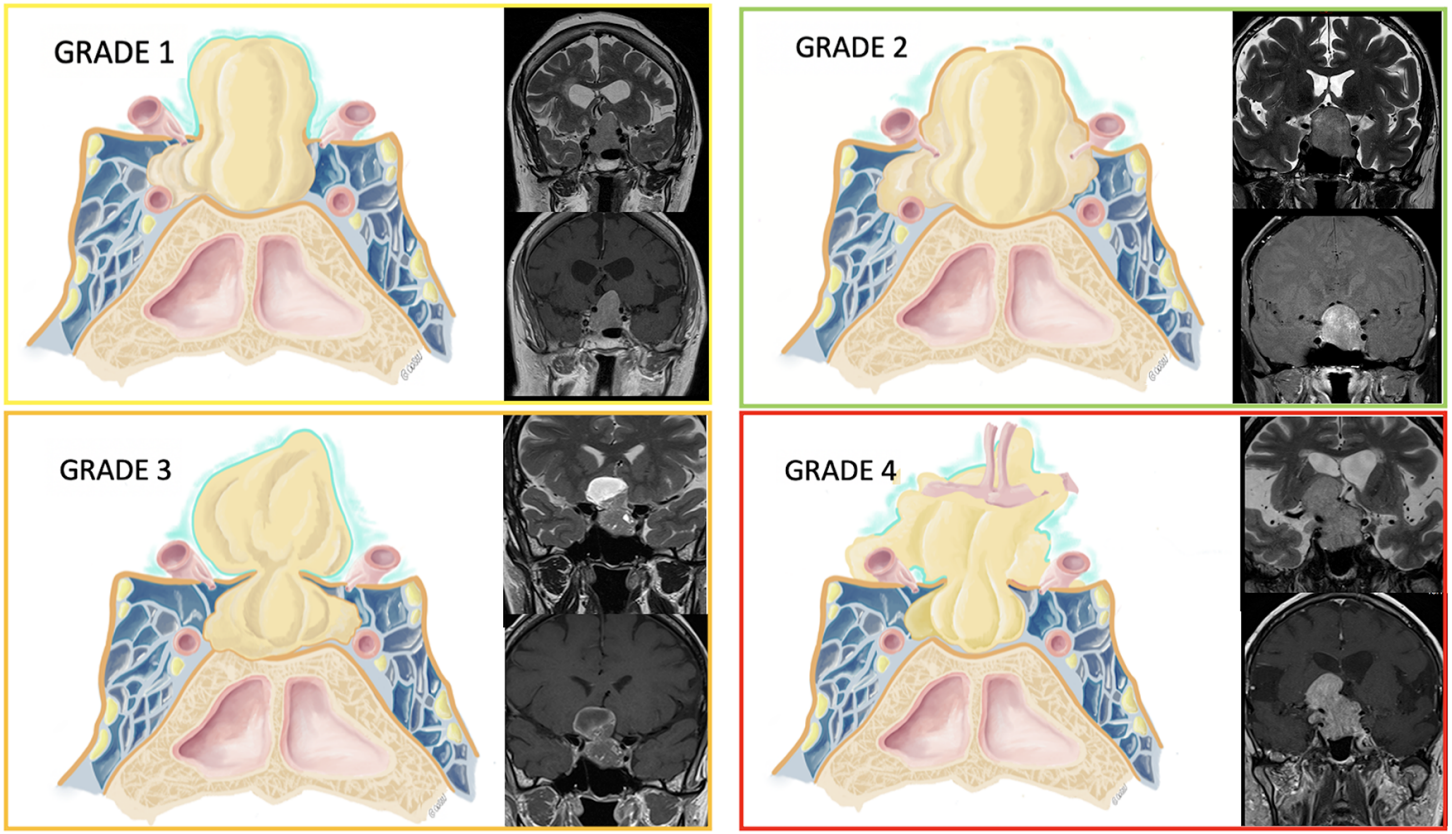
Tumors were classified according to their relationship with the diaphragm. A graphical representation is provided along with some examples of patients' MRI on the coronal plane (T2- and T1-weighted sequences after gadolinium injection).

We measured the size of the adenomas' cranio-caudal, medio-lateral, and antero-posterior diameters and defined the presence or absence of an invasion of the cavernous sinus through the application of Knosp classification. Tumors were classified as invading the cavernous sinus when the Knosp grade was >2, and the invasion of the medial wall of the cavernous sinus was confirmed during surgery. After the analysis of the preoperative MRI, we categorized the tumors according to their relationship with the diaphragm as follows (Figure 1):

• Grade 1: the adenoma extends to the suprasellar compartment through a wide incompetent diaphragm.
• Grade 2: the adenoma extends to the suprasellar compartment but stays infra-diaphragmatic due to a competent diaphragm.
• Grade 3: the adenoma extends to the suprasellar compartment despite a competent diaphragm through a small diaphragmatic opening resulting in a supra-diaphragmatic fragment (the typical dumbbell shape).
• Grade 4: the adenoma extends to the suprasellar compartment through a wide, incompetent diaphragm, invades the subarachnoid space, and becomes multilobulated with or without encasement of neurovascular structures.

In our surgical series, a classic endoscopic transsphenoidal surgery was performed using a uninostril approach. A medial transcavernous approach was performed to address the portion of the tumor invading the cavernous sinus. The transtubercular approach was performed for grade 3 or 4 tumors, to address the supradiaphragmatic portion and perform an extracapsular resection. When a transtubercular approach was planned, closure with a nasoseptal flap was performed to limit the risk of postoperative CSF leak. Transcranial surgeries were used in particular cases to complement the endonasal procedures in order to address extensions lateral to the internal carotid artery or when an invasion of the subarachnoid space was present, with encasement of neurovascular structures.

The surgical results were analyzed, and the extent of resection was classified as gross total resection (GTR) when no residual tumor was visible at 3-months postoperative MRI, near-total resection (NTR) when less than 5% of the initial tumor was left in place, and subtotal resection (STR) when a larger residual tumor was in place. Localization of postoperative residual tumor was also clearly defined.

All analyses were performed using the statistical software package STATA version 15 (College Station, TX, StataCorp LP). For categorical variables, *χ*^2^and Fisher's exact tests were performed. The significance level value was at *p* < 0.05.

## Results

A total of 37 patients with L-PAs or G-PAs were operated by one of our senior authors (MM and RTD) during the aforementioned period. There were 20 male patients (54%) and 17 female patients (46%). The median age at the time of surgery was 57 years (range 29–88).

There were 23 out of 37 patients who presented G-PAs (62%) while 14 had L-PAs (38%). In 34 out of 37 patients, a non-functional adenoma was identified (91.9%), in two cases a prolactinoma (5.4%), and in another case an ACTH-releasing macro-adenoma (2.7%).

The clinical and radiological data of our cohort are detailed in Table 1. Preoperative MRI image analysis revealed that the mean maximal diameter was 38.3 mm (±5.9 mm) (range 30 mm–50 mm). The invasion of the cavernous sinus was assessed during surgery. Patients with Knosp 3a showed no invasion of the cavernous sinus and only a lateral displacement of the medial wall of the cavernous sinus, which remained intact. Thus, invasion of the cavernous sinus occurred in 17 out of 37 patients (46%), while there was no invasion in 20 cases (54%). Sixteen cases were suprasellar with a supradiaphragmatic component and a large opening of the diaphragm (grade 1, 43.3%), ten cases were only infra-diaphragmatic (grade 2, 27%), and only two were supradiaphragmatic tumors with a narrow diaphragmatic opening (grade 3, 5.4%). Nine patients presented had a multi-lobular morphology with the invasion of the subarachnoid space (grade 4, 24.3%) (Table 1).

**Table 1:** Detailed clinical, pathological, and radiological data.

Mean age	57 years	29–88 years
Sex	Women	17 (46%)
	Men	20 (54%)
Clinical presentation	Visual disturbances	32 (86.5%)
	Headaches	12 (32.4%)
	Partial hypopituitarism	11 (29.8%)
	Total hypopituitarism	8 (22%)
	Secretory syndromes	3 (8.1%)
	Apoplexy	2 (5.4%)
	Incidental finding	1 (2.7%)
	Diabetes insipidus	0
Adenoma size	Large PAs	14 (38%)
	Giant PAs	23 (62%)
Immunohistochemistry	Non-functioning PA	34 (91.9%)
	• Gonadotroph	19
	• Silent	8
	• Null cell	7
	Functioning PA	3 (8.1%)
	• PRL secreting	2
	• ACTH secreting	1
Knosp grade	Knosp 0	1 (2.7%)
	Knosp 1	3 (8.1%)
	Knosp 2	5 (13.5%)
	Knosp 3a	11 (29.7%)
	Knosp 3b	6 (16.3%)
	Knosp 4	11 (29.7%)
Relationship with the diaphragm	Grade 1	16 (43.3%)
	Grade 2	10 (27%)
	Grade 3	2 (5.4%)
	Grade 4	9 (24.3%)

PA, pituitary adenoma.

**Silent:** non-functioning pituitary adenomas showing staining for a pituitary hormone at immunohistochemistry.

GTR was obtained in 7 patients (19%), NTR in 17 (46%), and STR in 13 (35%). All patients with GTR required no further surgery. Twelve patients underwent multiple surgeries to improve the extent of the resection, but none of them obtained GTR. Nine patients underwent a second endoscopic procedure where a classic transsphenoidal approach was performed when the residual tumor descended into the sella, while an extended endoscopic approach was performed to address the intracavernous part in the medial portion of the cavernous sinus or to address the suprasellar component through a transtubercular approach. Three cases underwent a transcranial approach: one for apoplexy of the residual tumor after endoscopic surgery and two for a lateral extension into the middle cranial fossa.

Invasion of the cavernous sinus had a significant impact on the extent of resection, as GTR was significantly higher in the cohort of patients with no invasion (7/20 patients with no CS invasion vs. 0/17 patients with CS invasion, *p* < 0.01). Table 2 summarizes the location of residual tumors in our cohort of 30 patients.

**Table 2:** Detailed surgical results.

Extent of resection	GTR	7 (19%)
	NTR	17 (46%)
	STR	13 (35%)
Second surgery		**TOT 12 pts**
	Endonasal endoscopic approach	9 (24%)
	Transcranial approach	3 (8%)
Localization of residual tumor		**TOT 30 pts**
	Middle cranial fossa	8 (27%)
	Cavernous sinus invasion	15 (50%)
	Posterior cranial fossa	1 (3%)
	Anterior cranial fossa	6 (20%)

GTR, gross total resection; NTR, near total resection; STR, subtotal resection.

**Figure 2:**
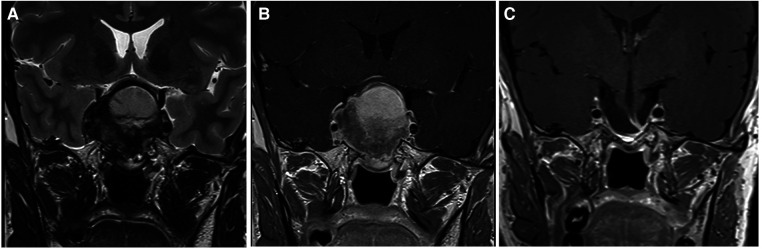
This large pituitary adenoma was classified as Knosp 3a and grade 1 according to the relationship with the diaphragm (Pictures A and B showing a coronal T2- and T1-weighted MRI after gadolinium administration, respectively). The diaphragm was wide open and the adenomas presented an oval shape. A gross total resection was possible through a classic endoscopic endonasal approach (Picture C), and no recurrent tumor is evident at 2 years of follow-up.

**Figure 3:**
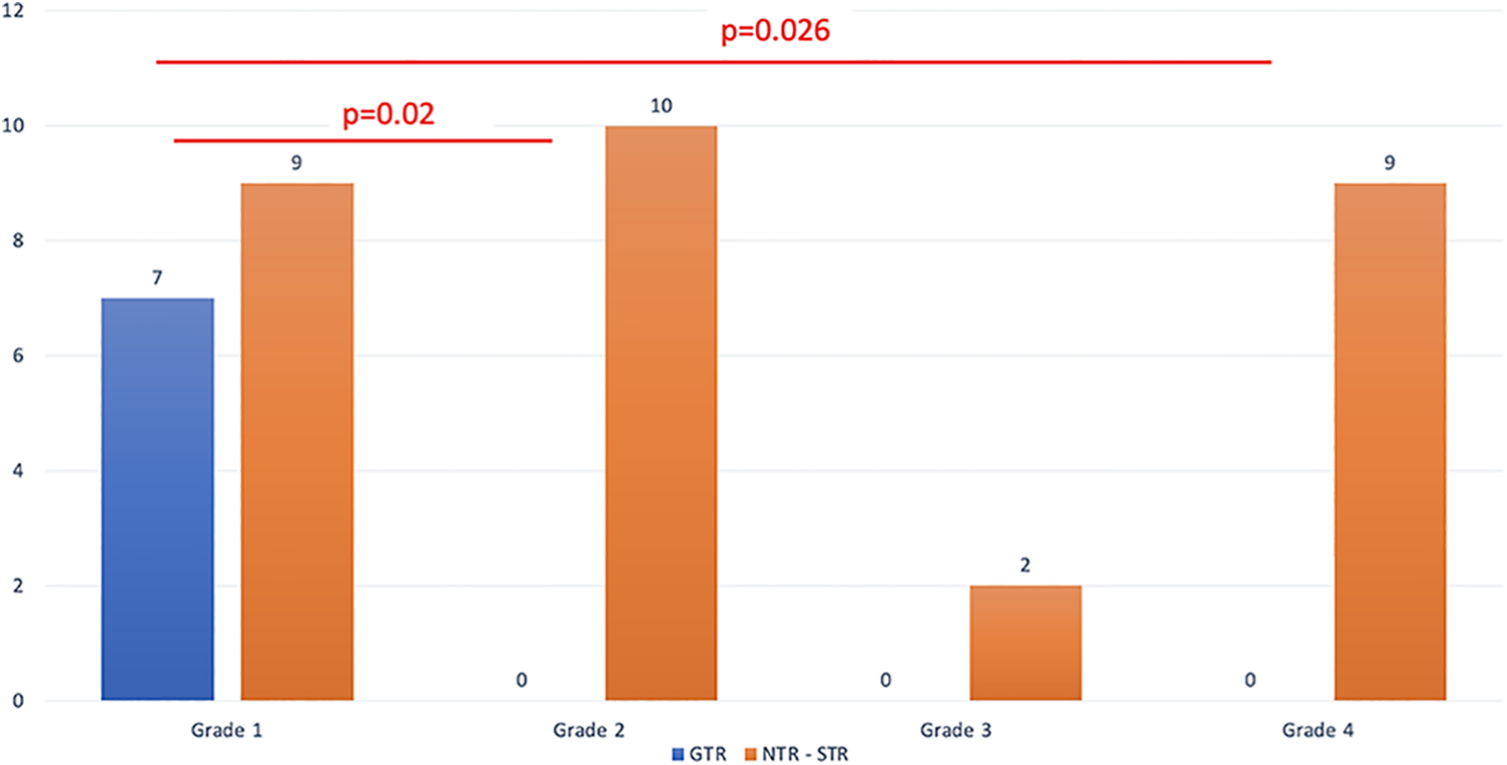
The extent of resection is detailed based on the relationship between the tumor and the diaphragm.

All of the patients where GTR was possible belonged to grade 1, that is, they had supradiaphragmatic extension with a large opening of the diaphragm (Figure 2). When the GTR rate was compared across the different grades, the difference was statistically significant (Figure 3).

**Figure 4:**
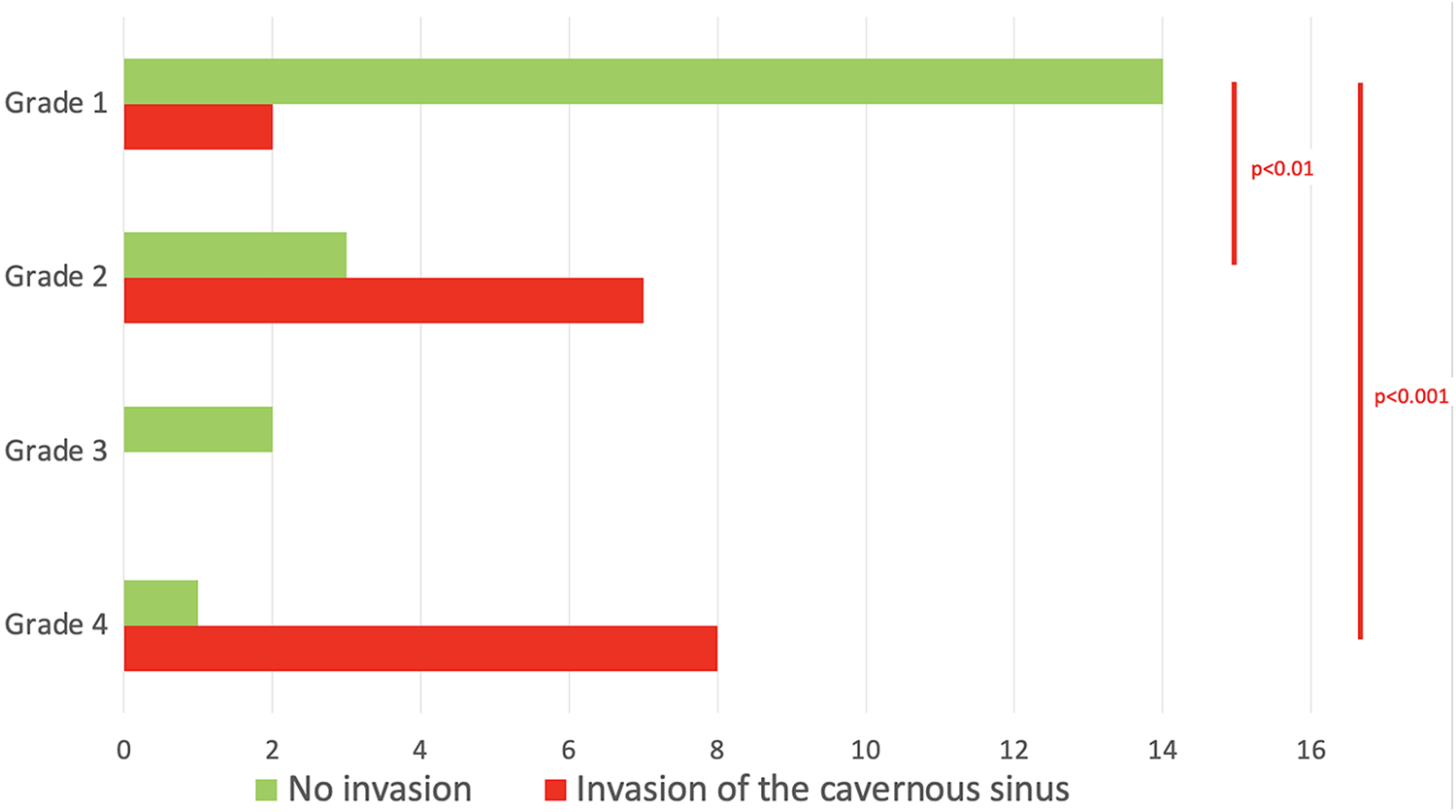
The invasion of the cavernous sinus is detailed for each grade.

GTR was not achieved in any of the patients with grade 2 tumor. This could be attributed to the fact that when the diaphragm is competent, large and giant tumors have a tendency to develop towards the weaker area, which is the medial wall of the cavernous sinus. This was confirmed by our analysis, where grade 2 tumors presented a higher rate of cavernous sinus invasion when compared with grade 1 (70% vs. 12.5% respectively, *p* < 0.01). As expected, for grades 3 and 4, where only STR or NTR were obtained, cavernous sinus invasiveness was high (8/11 cases, *p* = 0.003). The distribution of the invasion of the cavernous sinus, according to the different grades that we propose, is summarized in Table 3 and shown in Figure 4.

**Table 3:** The distribution of the different Knosp grades was detailed according to the relationship between the tumor and the diaphragm.

	Relationship with the diaphragm			
	1	2	3	4
Knosp grade				
Knosp 0	1	0	0	0
Knosp 1	3	0	0	0
Knosp 2	4	1	0	0
Knosp 3a	6	2	2	1
Knosp 3b	2	3	0	1
Knosp 4	0	4	0	7

**Figure 5:**
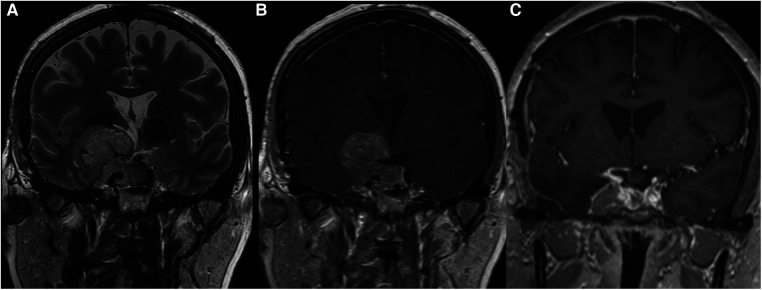
A giant macroadenoma showed invasion of the right cavernous sinus (Knosp 4), and it was classified as grade 4 according to the relationship with the diaphragm (Pictures A and B showing a coronal T2- and T1-weighted MRI after gadolinium administration, respectively). A combined approach through the use of a pterional approach and an endoscopic endonasal approach allowed to obtain a partial resection after addressing respectively the portion in the subarachnoid space and the sellar and suprasellar portion. The residual tumor in the right cavernous sinus (Picture C) was treated through Gamma Knife irradiation, and the tumor remained stable at 5 years of follow-up.

An example of the utility of a combined approach for a grade 4 tumor is reported in Figure 5.

The rate of surgical complications in this series was low. One patient had postoperative rhinorrhea and was treated with a second surgery (2.7%). Two patients had postoperative apoplexy and required trans-cranial surgery (5.4%). No new optic/oculomotor nerve palsies occurred following surgery in this series.

## Discussion

Surgical series dealing with L-PAs and G-PAs reported GTR in less than 50% of cases after a single surgical procedure (3, 11, 12, 17, 25). The dimensions of the tumor are not the main limiting factor in the performance of GTR; the shape seems to play an important role, as dumbbell-shaped and multilobulated PAs are respectively associated with decreased GTR, varying from 82% for round and oval PA to 0% for multilobulated PA (17, 20). Rather, it is universally accepted that the factors that preclude a complete resection are the hard consistency of the tumor, the invasion of the cavernous sinus. and the the invasion of the subarachnoid space (10, 11, 17, 21, 26).

Few classifications focused on G-PAs. One of the first was Goel's classification, which divided tumors into four grades according to the invasion and elevation of the roof of the cavernous sinus and the neurovascular encasement in the subarachnoid space (5). According to this vision, the diaphragm is in general stretched and elevated on the dome of the tumor. Recently, another classification was proposed, based on the antero-posterior, infero-superior, and lateral extensions (27). These classifications are based on tumor extension, and we agree with Micko et al. (28) that one important limiting factor for tumor resection is the neck to dome ratio, determining the feasibility of an endonasal approach for these tumors and the surgical nuances of the approach necessary to achieve a maximal resection, such as the section of the diaphragm. For tumors with large suprasellar extensions, it is well known that the diaphragm can be distended and displaced markedly in a superior direction above the tumors, even up to the third ventricle (5), but true dumbbell shape tumors are caused by a low diaphragm with a narrow opening. The anatomy of the diaphragm determines the manner in which the endonasal surgery is performed, and it dictates when a transtubercular approach is necessary to access the large supradiaphragmatic component or when the incision of the diaphragm is required to have trans-diaphragmatic access.

Not much has been published about the conditions associated with a competent or incompetent diaphragm (22, 30), and some authors highlighted the role of intracranial hypertension and CSF dynamics (34–37). Campero et al. performed an anatomical study and classified their specimens into three groups according to the diaphragm opening: group A (<4 mm), group B (4–8 mm), and group C (>8 mm), assuming that the anatomic variability of the diaphragm opening, along with the morphology of the medial wall of the cavernous sinus, may explain the pattern of growth of pituitary tumors (22).

Thus, we studied the role of the diaphragm in determining the shape of Pas, as well as the relationship between the classification we proposed and the extent of resection performed. In group 1, when the diaphragm is large offering a natural passage to adenoma growth, the best results in terms of the extent of resection were achieved. All the tumors where GTR was possible in one single procedure could be categorized in group 1. This result was statistically significant compared with the other morphologies. Indeed, when the diaphragm is wide open, tumor resection through an endoscopic endonasal approach may be safely performed as a large working corridor is present, and the pulsating effect of CSF during surgery or specific maneuvers to increase the intracranial pressure may help in the descent of residual tumor (28, 29). The value of endoscopic procedures to address these tumors was also addressed by Jin et al., and the authors reported an elevated rate of GTR (90%) (30).

At the same time, the absence of cavernous sinus invasion was associated with a higher rate of GTR, suggesting that the GTR observed in group 1 is dependent on parasellar extension and Knosp grade. Cavernous sinus invasion is a well-described limiting factor in the resection of sellar lesions, in particular when it extends lateral to the cavernous portion of the carotid artery (31, 32). We therefore could say that a higher rate of GTR can be associated with the morphology in group 1 for L-PAs and G-Pas and that this is higher when CS invasion is absent.

To summarize, three factors must be considered when planning the surgical procedure: the shape and tumor relationship to the diaphragm (proposed classification), the invasion of the cavernous sinus, and the hormonal secretion of the adenoma.

For grades 1 and 2, we performed a classic endoscopic approach. GTR was obtained as expected in a high proportion of patients with grade 1 tumors. However, in group 2, GTR was not achieved in any of the patients. This was related to the fact that when a large adenoma expands, it remains restricted superiorly by a competent diaphragm, and therefore, the tumor expands towards a zone of relatively less resistance called the infrasellar or parasellar space, through the medial wall of the cavernous sinus. In these cases, the limiting factor in obtaining GTR is the cavernous sinus invasion, and extended transcavernous approaches may be advised to obtain a greater resection.

In grade 3, the driving force of tumor growth pushes the adenoma through a small opening in a competent diaphragm, creating the typical dumbbell-shaped tumor. With this morphology, the supradiaphragmatic portion of the adenoma can be more difficult to access through a classic transsphenoidal endoscopic approach, as previously described. To obtain a greater extent of resection, a transtubercular extended transsphenoidal approach or a trans-diaphragmatic approach with incision of the diaphragm can be chosen (11). This procedure however requires a careful reconstruction of the skull base.

For multilobulated adenoma invading the subarachnoid space or the middle or anterior cranial fossae (grade 4), GTR through one single approach is extremely challenging (13–15). The complexity of surgery is secondary to the encasement of neurovascular structure in their cisternal portion and the lack of a well-defined tumor capsule (13, 14). A combination of transcranial and endoscopic endonasal techniques should thus be considered (13, 16, 33). The timing between the two approaches depends on the size of the suprasellar extension.

Besides our classification and the invasion of the cavernous sinus, the hormonal secretion of the adenoma should be taken into consideration, as more aggressive approaches should be performed to obtain a biological remission with secreting tumors.

Experienced skull base surgeons and expertise in a tertiary care center are necessary when dealing with large, giant, invasive tumors, and a careful analysis of the relationship existing between the tumor and the diaphragm is mandatory to plan the surgery and predict the extent of resection after the procedure.

### Limitations

Because this is a retrospective analysis of a tertiary care pituitary center about a rare pathology, we report a small surgical cohort, and the power of our statistical analysis may be limited, with no multivariate analysis possible. We were unable to determine the role of tumor consistency in predicting the extent of resection because, in order to perform a proper analysis of this factor, we need to exclude all the adenomas with CS invasion. Unfortunately, in our series, the power of our analysis was strongly reduced by the limited number of L- and G-PAs with no CS invasion.

Another limitation is represented by the fact that this proposed classification is based on subjective careful preoperative analysis rather than specific morphometric criteria.

Larger multicentric studies are mandatory to have an external validation and confirmation of the clinical and surgical relevance of our proposed grading system.

## Conclusion

L-PAs and G-PAs are rare diseases in which GTR remains challenging. Careful radiological evaluation of the relationship between the tumor and the sellar diaphragm, and invasion of the cavernous sinus or the subarachnoid space are crucial factors in determining the surgical strategy and the possibilities of achieving gross total resection. When a wide opening of an incompetent diaphragm is present without invasion of the cavernous sinus, GTR is commonly achieved. In all other morphologies, GTR is more difficult to obtain, so extended endoscopic or combined approaches need to be considered.

In the drawings, the diaphragm is represented in orange, while the arachnoid is represented as a teal thinner layer.

• Grade 1: The adenoma is extending in the suprasellar compartment, and the diaphragm is wide open. As shown in the MRI, the diaphragm is not visualized on the dome of the tumor, which is only covered by a thin layer of arachnoid.
• Grade 2: The pituitary adenoma is extending in the suprasellar compartment, but stays infra-diaphragmatic (the diaphragm is competent). The diaphragm is visible as a thin hypointense line on T2-weighted sequences, and it delimitates the dome of the tumor.
• Grade 3: The adenoma is extending in the suprasellar and supradiaphragmatic compartment with a competent diaphragm, giving a typical dumbbell shape to the tumor. The MRI shows a pituitary adenoma with a component in the suprasellar space. The opening of the diaphragm is narrow, thus limiting the access to the suprasellar component during a standard transsphenoidal approach.
• Grade 4: The adenoma is extending to the suprasellar compartment, it is multilobulated and invades the subarachnoid space, with a rupture of the arachnoid membrane (thin teal layer in the drawing) and a possible encasement of nervous and vascular structures (such as the anterior communicating complex as shown in the picture). The pituitary MRI shows a tumor with complex morphology, and the hypersignal of the mesial temporal lobe is evident on T2-sequences, witnessing an invasion of the subarachnoid space.

## Data Availability

The raw data supporting the conclusions of this article will be made available by the authors, without undue reservation.
