# Erratum: Relationship with the diaphragm to predict the surgical outcome in large and giant pituitary adenomas

**DOI:** 10.3389/fsurg.2023.1176644

**Published:** 2023-03-14

**Authors:** Frontiers Production Office

**Affiliations:** Frontiers Media SA, Lausanne, Switzerland

**Keywords:** cavernous sinus invasion, diaphragm, endoscopy, pituitary adenoma, surgery

An Erratum on Relationship with the diaphragm to predict the surgical outcome in large and giant pituitary adenomas By Harel E, Cossu G, Daniel RT and Messerer M. (2022) Relationship with the diaphragm to predict the surgical outcome in large and giant pituitary adenomas. Front. Surg. 9:962709. doi: 10.3389/fsurg.2022.962709

An omission to the funding section of the original article was made in error. The following sentence has been added: “Open access funding was provided by the University of Lausanne”.

The original version of this article has been updated.

